# Tremor and clinical fluctuation are related to sleep disorders in Chinese patients with Parkinson’s disease

**DOI:** 10.1186/2047-9158-3-21

**Published:** 2014-10-22

**Authors:** Hongyan Zhou, Cunzhou Shen, Jie Chen, Hao Qian, Yifan Zheng, Yanmei Liu, Wenbiao Xian, Zhong Pei, Ling Chen

**Affiliations:** 1 Department of Neurological Intensive Care Unit, The First Affiliated Hospital of Sun Yat-sen University, Guangzhou 510080, China; 2 Department of Internal Medicine, Nansha central Hospital, Guangzhou 511457, China; 3 Department of Neurology, The Second Affiliated Hospital of Guangdong Pharmaceutical University, Guangzhou 510300, China; 4 Department of Neurology, The First Affiliated Hospital of Sun Yat-sen University, NO. 58 Zhongshan Road 2, Guangzhou 510080, China

**Keywords:** Sleep disorders, Parkinson’s disease, Parkinson’s disease sleep scale-Chinese Version(PDSS-CV), Unified Parkinson’s Disease Rating Scale (UPDRS)

## Abstract

**Objective:**

To study the relationship between sleep disturbances and symptoms in patients with Parkinson’s disease (PD).

**Methods:**

The Parkinson’s Disease Sleep Scale-Chinese Version (PDSS-CV) was used to evaluate the sleep disturbances of PD patients in a cross sectional study. The Unified Parkinson’s Disease Rating Scale (UPDRS) parts II-IV, and the Hoehn & Yahr (H&Y) stage were used to determine the level of motor function in PD and the severity of PD. The Spearman correlation and a multiple regression analysis were used to identify the relationship between sleep disturbances and symptoms of PD. The quantities derived from the UPDRS and the H&Y stage and disease duration were compared between groups of patients either with or without sleep disturbances identified by the PDSS. This study was conducted from December 2011 to March 2012 at the First Affiliated Hospital of Sun Yat-sen University, in Guangzhou.

**Results:**

A total of 136 PD patients were included in this study. The overall total PDSS score in PD patients was 107.58 ± 23.35 points (range: 30–146). There were significant differences in the disease duration, the H&Y stage, and the UPDRS section subscores between groups of patients either with or without sleep disturbances (Kruskal-Wallis Test, *p* <0.05). There were significant negative correlations between PDSS scores and the UPDRS subscores, the H&Y stage and the disease duration (Spearman correlation, *p* < 0.05). The multiple regression analysis indicated that sleep disturbances identified by the PDSS were only associated with daily life activity, tremor intensity and clinical fluctuation (R^2^ = 0.22, F(3,132) = 12.4, *p* < 0.001). The correlations were also significant when the contribution of the other two factors was excluded using partial correlations.

**Conclusions:**

The level of daily life activity and the occurrences of tremor and clinical fluctuation are likely to be important factors that lead to PD patients’ sleep disturbances. This study may elucidate an important clue for the relationship between sleep disturbances and PD symptoms.

## Introduction

Parkinson’s disease (PD) is the second most common neurodegenerative disorder in the world. This disease is characterized by both motor dysfunction and non-motor symptoms such as sleep problems and mood abnormalities. Community studies have reported that 60-98% of PD patients have a sleep disorder [1]. One study showed that the prevalence of PSG-quantified sleep disturbances in a multi-ethnic Asian population with PD was 81% [2]. The prevalence of sleep disturbances is high, yet the occurrence of active complaints in the clinic is uncommon, suggesting that sleep disturbances are often under-recognized and do not receive adequate treatment in PD patient in China.

PD is one of many incurable chronic disorders, so the aim of therapy in these patients is to improve their quality of life (QoL). Neuropsychiatric symptoms, especially depression, nighttime sleep disorders, motor fluctuations, and unpredictable fluctuations are the variables that most affect the QoL of patients with PD [3]. These variables may interact with each other. The RECOVER trial showed that treatment with rotigotine in PD patients resulted in significant benefits in control of both motor function and nocturnal sleep disturbances [4]. Some PD patients reportedly experience ‘sleep benefit’: an improved motor functioning upon awaking in the morning [5,6]. Previous studies have indicated that PD symptoms may contribute to sleep disorders [7,8]. The treatment of PD with levodopa preparations can improve sleep quality in PD patients [9]. Recent studies demonstrated that individual suffering from rapid eye movement (REM) sleep behavior disorder (RBD) carry a high specific risk (up to 80%) for developing a neurodegenerative disorder of α- synucleinopathy (e.g. Parkinson’s disease, dementia with Lewy bodies and multiple system atrophy) within 10–20 years [10]. These results show that there are some associations between PD motor symptoms and sleep disturbances. The identification of sleep disturbances and impaired motor function or complications of therapy will help us to improve our treatment approach to achieve the main goal of improving the QoL of our patients.

The Parkinson’s Disease Sleep Scale (PDSS) was proposed by Chaudhuri and his colleagues [11]. This scale is a disease-specific instrument that can quantify the most common sleep problems in PD patients; the PDSS can also identify sleep disturbances that had not been previously diagnosed, such as sleep-maintenance insomnia and excessive daytime sleepiness [12]. The PDSS provides an objective method for assessing sleep quality in PD patients across countries with different cultures [13]. This bedside tool has been translated into simplified Chinese and validated in China [12].

In the present study, the relationships between sleep disturbances and impaired motor function and complications of therapy were investigated using the Chinese version of the PDSS and the UPDRS in Chinese PD patients.

## Patients and methods

This is a cross-sectional study. A total of 136 consecutive PD patients (85 male, 62.5%) participated in this survey in our hospital during the period from December 2011 to March 2012. The diagnoses of PD were based on the UK Parkinson’s Disease Society Brain Bank clinical diagnostic criteria [14]. Any patients receiving deep brain stimulation or psychotropic treatments or presenting with dementia, Parkinsonism, progressive supranuclear palsy, multiple system atrophy or other psychiatric diseases were excluded. This study was approved by the Ethic Committee of the First Affiliated Hospital, Sun Yat-sen University, and informed consent was obtained from all of the participants.

The sleep disturbances were evaluated using the Chinese version of the PDSS [12]. The PDSS was used to evaluate the patients’ experiences over the previous week. The PDSS consists of 15 items and reports the overall quality of a night’s sleep (item 1), sleep onset and maintenance insomnia (items 2 and 3), nocturnal restlessness (items 4 and 5), nocturnal psychosis (items 6 and 7), nocturia (items 8 and 9), nocturnal motor symptoms (items 10–13), sleep refreshment (item 14), and daytime dozing (item 15) [11]. The possible score for each item ranges from 0 (symptoms are severe and constant) to 10 (symptom-free). The maximum possible cumulative score for the PDSS is 150. Scores of 105 or higher suggest normal sleep, and scores of 90 or less suggest the presence of sleep disturbances [15]. A score of less than five on any item indicated a severe sleep disturbance [16].

The Unified Parkinson’s Disease Rating Scale (UPDRS) is a widely used clinical tool that assesses functional status and motor performance. To analyze the possible associations between the characteristics of PD and sleep problems, the following clinical measurements were taken: the level of daily life activity (UPDRS part II); the occurrence of motor-symptom subgroups, such as tremor (UPDRS part III, questions 20 and 21), rigidity (question 22), bradykinesia (questions 23–26 and 31), gait and postural stability (questions 27–30), bulbar abnormalities (questions 18 and 19); and motor complications of therapy, such as motor fluctuation (questions 36–39) and dyskinesia (questions 32–34) [17].

To compare the efficacies of different medicines directly, the daily L-dopa equivalent dose(LED) was calculated on the basis of the following equivalences: 100 mg of standard L-dopa equals 140 mg of controlled release L-dopa, 10 mg of selegiline, 1 mg of pramipexole, 1 mg of pergolide, 10 mg of bromocriptine, and 100 mg of piribedil. Then, the summed standard levodopa dose and levodopa controlled release dose were multiplied 0.25 times more if the patient was taking entacapone [18,19].

The data were analyzed using SPSS 17.0 for Windows (Chicago, IL). The total mean scores for the PDSS were calculated. Correlations between PDSS scores and the following variables: age, the H&Y stage, the disease duration, the level of daily life activity, and the occurrences of motor symptoms and drug complications, were examined. A multiple linear regression analysis was used to evaluate the contribution of each factor for sleep disturbances using a stepwise method. The variables which were significantly associated with sleep disturbances were compared in the group of patients split in three classes according to a cut-off value for PDSS (PDSS ≤90, PDSS 91–104, PDSS ≥105) using the Kruskal-Wallis Test. A significance level of 0.05 was chosen.

## Results

The age of the patients was 64.21 ± 11.48 years old, and the disease duration was 5.98 ± 5.04 years. The severity of PD was as follows: H&Y stage, 2.15 ± 0.82; UPDRS scores (parts II to IV), 42.51 ± 23.26.

The total PDSS score in PD patients was 107.58 ± 23.35 points (range: 30–146). Overall, 26 patients (19.1%) had sleep disturbances (a PDSS score of less than 90), 86 patients (63.2%) had normal sleep (a PDSS score of more than 105), and 24 patients (17.6%) had possible sleep disturbances (a PDSS score between 90 and 105). The prevalence of sleep disturbances identified by PDSS increased with the disease severity (Table 1): 3 out of 28 patients (10.7%) when H&Y = 1; 8 out of 68 patients (11.8%) when H&Y = 2; 11 out of 31 patients (35.5%) when H&Y = 3; 4 out of 9 patients (44.4%) when H&Y = 4. Nocturia (item 8, 55 out of 136 patients (40.4%), mean, 4.98 ± 3.70), daytime sleep (item 15, 40 out of 136 patients (29.4%), mean, 5.9 ± 3.08) and sleep maintenance insomnia (item 3, 40 out of 136 patients (29.4%), with a mean, 6.04 ± 2.30) were the three most severe symptoms that were reported to cause sleep disturbances.

**Table 1:** The prevalence of sleep disturbances identified by the PDSS with different disease severity

**H&Y stage**	**H&Y = 1**	**H&Y = 2**	**H&Y = 3**	**H&Y = 4**	**total**
**PDSS score**					
<90	3	8	11	4	26
PDSS group %	11.5%	30.8%	42.3%	15.4%	100.0%
H&Y stage %	10.7%	11.8%	35.5%	44.4%	19.1%
90 ~ 105	2	13	7	2	24
PDSS group %	8.3%	54.2%	29.2%	8.3%	100.0%
H&Y stage %	7.1%	19.1%	22.6%	22.2%	17.6%
>105	23	47	13	3	86
PDSS group %	26.7%	54.7%	15.1%	3.5%	100.0%
H&Y stage %	82.1%	69.1%	41.9%	33.3%	63.2%
total	28	68	31	9	136
PDSS group %	20.6%	50.0%	22.8%	6.6%	100.0%
H&Y stage %	100.0%	100.0%	100.0%	100.0%	100.0%

The differences in the disease duration, the H&Y stage, the UPDRS scores and subscores were significant between groups of patients either with or without sleep disturbances identified by PDSS (Kruskal-Wallis Test, *p* <0.05), whereas the differences in bradykinesia and dyskinesia were not (Table 2). The patients with more marked sleep disturbances had longer disease durations, more severe PD symptoms (as measured by H&Y stage), and higher UPDRS scores.

**Table 2:** T**he differences of the disease duration, the H&Y stage, the L-dopa equivalent dose, and the UPDRS scores and subscores among PDSS-identified sleep disturbances**

**items**	**Groups**					
	**Score ≤ 90 (n = 26)**		**91 ~ 104(n = 24)**		**≥105(n = 86)**	
	**Mean (SD)**	**Min-Max**	**Mean (SD)**	**Min-Max**	**Mean (SD)**	**Min-Max**
**Male. n (%)**	15(17.6)		15(17.6)		55(64.7)	
**Age at visit, years**	63.42(2.68)	34-87	70.42(1.48)	51-88	62.71(1.22)	29-80
**Age at onset, years**	54.21(2.84)	27.5-81	64.54(1.97)	40-81	57.68(1.27)	27.5-78
Disease duration**	9.21(6.14)	0.5-27	5.88(5.59)	1-27	5.03(4.09)	0.5-20
H&Y stage**	2.62(0.90)	1-4	2.38(0.77)	1-4	1.95(0.75)	1-4
Levodopa*	494(208)	100-950	339(233)	0-786	360(261)	0-1196
UPDRS score**	56.19(29.93)	19-141	48.46(26.75)	18-121	36.72(17.36)	10-111
Daily life activity*	16.85(8.68)	6-41	14.96(8.93)	6-37	11.44(5.11)	3-33
Motor function**	36.27(21.06)	3-91	32.33(18.27)	10-83	24.00(12.30)	4-73
Tremor*	5.96(5.42)	0-20	5.58(3.87)	0-15	3.53(3.05)	0-14
Rigidity**	8.46(4.55)	0-19	6.17(4.58)	0-17	5.26(3.44)	0-15
bradykinesia	13.54(8.58)	1-30	12.04(8.61)	1-36	9.27(6.23)	0-36
Gait/postural stability*	5.23(3.73)	0-15	5.38(3.82)	0-16	3.61(2.95)	0-16
Bulbar abnormalities**	3.08(1.35)	1-7	3.17(1.63)	0-8	2.33(0.99)	0-5
Complication**	3.08(2.62)	0-9	1.17(1.09)	0-4	1.28(1.97)	0-11
Motor fluctuation**	1.46(1.96)	0-7	0.04(0.2)	0-1	0.44(1.06)	0-4
Dyskinesia	0.50(1.03)	0-4	0.17(0.64)	0-3	0.26(0.84)	0-6

*:*p* < 0.05; **:*p* < 0.01. Kruskal-Wallis Test, max = maximum; min = minimum.

There were significant negative correlations between the PDSS scores and the disease duration (r = -0.298, *p* <0.001), the H&Y stage (r = -0.308, *p* <0.001) and all UPDRS subscores, including the level of daily living activity (UPDRS II, r = -0.290, *p* =0.001), and the occurrences of motor dysfunction (UPDRS III, r = -0.234, *p* =0.006) and complication (UPDRS IV, r = -0.436, *p* <0.001). A univariate regression analysis showed that the PDSS score was correlated with all of the examined parameters (Table 3).

**Table 3:** A univariate analysis of the factors associated with the PDSS scores

**Variable**	**Regression coefficient**	**95% CI**	**Standardized coefficient**	**t-score**	***p*-value**	**R**^**2**^
Disease duration	-1.346	-2.103 to -0.589	-0.291	-3.517	0.001	0.085
HY stage	-9.57	-14.125 to -5.016	-0.338	-4.156	0.000	0.114
levodopa	-0.016	-0.031 to 0.000	-0.171	-2.004	0.047	0.029
Daily life activity	-1.274	-1.8 to -0.747	-0.382	-4.785	0.000	0.146
Tremor	-1.994	-2.965 to -1.024	-0.331	-4.065	0.000	0.11
Rigidity	-1.918	-2.848 to -0.988	-0.332	-4.08	0.000	0.11
Bradykinesia	-0.913	-1.487 to -0.338	-0.262	-3.142	0.002	0.069
Gait/postural stability	-2.009	-3.15 to -0.868	-0.288	-3.481	0.001	0.083
Bulbar abnormality	-4.812	-7.902 to -1.721	-0.257	-3.079	0.003	0.066
Motor fluctuation	-5.135	-8.124 to -2.146	-0.282	-3.398	0.001	0.079
Dyskinesia	-3.18	-7.385 to 1.476	-0.116	-1.351	0.179	0.013

The regression coefficient is an unstandardized beta weight and the standardized coefficient is a beta weight as well; CI = confidence interval.

We ran a regression model that used the PDSS score as the dependent variable, and the H&Y stage, the disease duration, the levodopa dosage, the daily life activity, motor symptoms and drug complications as independent variables. A multiple regression analysis showed significant effects for only the level of daily life activity and the occurrences of tremors and clinical fluctuation (R^2^ = 0.22, F(3,132) = 12.4, *p* < 0.001) (Table 4). The other variables did not correlate with the PDSS scores, therefore, they were excluded from the model. Furthermore, partial correlations indicated that significant correlations were found between the PDSS scores and each one of the above three factors (the level of daily life activity, the occurrences of tremors and clinical fluctuation) when the contributions of the two other variables were excluded (*p* <0.05).

**Table 4:** **A multiple regression analysis of the factors associated with the PDSS scores (R**^**2**^ **= 0.22,F(3,132) = 12.4,** ***p*** **< 0.001)**

**Variable**	**Regression coefficient**	**95% CI**	**Standardized coefficient**	**t-score**	***p*-value**
Daily life activity	-0.716	-1.325 to -0.107	-0.215	-2.325	0.022
Tremor	-1.346	-2.41 to -0.281	-0.224	-2.501	0.005
Motor fluctuation	-4.131	-7.023 to -1.239	-0.227	-2.826	0.005

CI = confidence interval.

## Discussion

To our knowledge, this study is the first to estimate the correlation between sleep disturbances identified with the PDSS and the PD symptoms evaluated by the UPDRS.

It has been shown that the PDSS is a good scale for quantifying sleep disturbances in PD patients, and it is becoming used increasingly in the PD field in Asia because of its excellent test–retest reliability and easy application [2,12,20]. Thus, the PDSS was used to identify sleep disturbances in the present study. The total PDSS score in PD patients was 107.58 ± 23.35 points, compatible with other cross-sectional study using PDSS [3,12,16,21,22]. Scores of 90 or less suggest the presence of sleep disturbances, according to Porter’s study [15], the frequency of sleep disturbances detected by the PDSS in the current study was 20%, which was similar to Porter’s study [15].

In the present study, we found that the PDSS-identified sleep disturbances correlated with all the examined parameters. Interestingly, further study using multiple regression analysis revealed that only the level of daily life activity and the occurrences of tremor and clinical fluctuation had stable associations with the PDSS scores. The correlations were also significant even when using partial correlations excluding the contribution of the other two factors.

The PDSS score correlated with the daily life activity score. Correlations between sleep disturbances and daily life activity have been reported previously for various diseases. For example, in depressed patients, insomnia is associated with poor health-related quality of life. The treatment of insomnia in depressed patients produced superior improvement in the health-related quality of life [23]. In dementia patients, sleep disturbances were also associated with decreased daily activity [24]. Our results supported this idea by showing that the prevalence of sleep disturbances identified by the PDSS increased with the disease severity (Table 1). Research has revealed that impaired bed mobility seemed to be the reasons that the sleep disturbances correlated with daily life activity. Stack & Ashburn [7] evaluated the sleep of 38 people with PD and observed their turning strategies and found that most reported difficulty maintaining sleep and difficulty turning. Using multiple strategies they found impaired bed mobility was associated with sleep disturbance [7]. Recently Louter’s study also found that subjectively impaired bed mobility in Parkinson disease affects sleep efficiency [8]. Therefore, treatment strategies designed to improve bed mobility may help improve sleep quality.

The PDSS score was associated with tremor but not with other PD motor symptoms. Motor symptoms such as tremor, rigidity, and bradykinesia have been shown to cause sleep fragmentation [25], whereas improving motor function in PD patients can alleviate some sleep problems [20,26]. The sleep disturbances resulting from the appearance of PD symptoms can occur during any sleep stage, but they are most common in light sleep [27]. Patients with PD may be more sensitive to tremor than other symptoms during sleep. Tremor is associated with awakenings, microarousals, body movements and sleep-state changes [25]. In the light-sleep stage, tremor may affect the comfort of PD patients, induce frequent and prolonged awakenings, disrupt sleep continuity, and cause sleep fragmentation. PD patients may complain of an inability to fall back to sleep. The relationship between the PDSS score and tremor suggested that tremor, but not the other motor symptoms, likely plays the most critical role in PD sleep disorders.

In our study, sleep disturbances were correlated with motor fluctuation during sleep. A logistic regression analysis with sleep quality as the dependent variable revealed that the on/off phenomena is associated with sleep disturbances in PD patients [28]. Patients with PD may have difficulties turning over in bed in the unpredictable “off” state and difficulties turning over in bed are one cause of nocturnal awakening [29].

The level of medication is an important factor for sleep disturbance in PD [30,31]. In the present study, we used the levodopa equivalent dose(LED) to compare the efficacies of different medicines directly. Surprisingly, in the multiple regression analysis using the PDSS scores as the dependent variable, only activity of daily living, tremor and clinical fluctuations were significantly correlated with sleep disturbance. In cross-sectional studies, the relationships between sleep disturbances and dopaminergic medications are not clear. Our result is similar with Pellecchia’s study in Italian PD patients [21]. Sleep quality in patients who took dopaminergic agonists did not differ from that of patients who took levodopa in monotherapy [32]. Stefansdottir’s study [33] also shows only minor changes in PDSS scores in PD patients before and after the introduction of dopaminergic treatment (levodopa, dopamine agonists or MAO-B inhibitor alone or in combination with one of the other). Changes in PDSS total and subdomain scores from baseline to follow-up did not differ between the levodopa and dopamine agonist groups [33]. Influence of medication on sleep in PD is complex and drug treatment itself may have either negative or positive impact on sleep quality [34]. Lower doses of dopamine agonists can improve sleep quality partly by reducing motor symptoms [35,36]. However, the same drugs may cause insomnia and excessive daytime sleepiness [37]. Our result suggests that the medications’ effects on sleep disturbances were non-significant, at least in this study.

There are some limitations in this study. First, disease duration was not included in selection criteria and this is a limitation because disease duration is critical to properly diagnose PD. Accuracy for PD diagnosis in patients with less than 5 years of disease is about 26-53% [38]. All cases in this study were examined by a group of movement disorder specialists and had improvement when treated with dopaminergic medications, which may improve the accuracy of PD diagnosis. Second, depression was not assessed in this study. The relationship among depression, sleep disorders and PD is very complicated. Depression can affect sleep and is prevalent among PD patients [34]. Major depressive disorder been associated with an increased risk of subsequent PD [39]. One study aiming to quantify sleep problems in PD patients shows antidepressive drugs had no effect on the scores obtained through the PDSS scale [32]. In the future, we should consider the effect of depression when designing a new study about sleep disorders in PD patients. Third, a properassessment of specific medications that could affect sleep was not performed. However, as discussed on the above paragraph, using of levodopa equivalent dose(LED) limits potential impact of other medications on sleep in this study also can provide helpful information for us in some degree.

## Conclusions

In this study, approximately 20% of PD patients were found to be suffering from sleep disturbances. The significant correlations between sleep disorders and the variables of the level of daily life activity and the occurrences of tremor and clinical fluctuation (but not with disease duration, H&Y stage, levodopa and all the other PD symptoms) suggests that daily life activity, tremor and clinical fluctuation are likely to be important factors that lead to PD patients’ sleep disturbances. This study provides important information about the relationship between sleep disturbances and PD symptoms.

## Abbreviations

PD: Parkinson’s disease; PDSS: Parkinson’s Disease Sleep Scale; UPDRS: The Unified Parkinson’s Disease Rating Scale; H&Y: Hoehn&Yahr; LED: Levodopa equivalent dose; QoL: Quality of life; RBD: Rapid eye movement (REM) sleep behavior disorder.

## Competing interests

The authors declare that they have no competing interest and have approved the final article.

## Authors’ contributions

HYZ, JC, CZS, HQ, YFZ performed the research; YML, WBX participated in the design of the study and performed the statistical analysis. HYZ, LC, ZP prepared the manuscript. All authors read and approved the final manuscript.
